# Associations Between Mental Health and Oral Health in Saudi Arabia: An Online Survey-Based Cross-Sectional Study

**DOI:** 10.7759/cureus.31732

**Published:** 2022-11-21

**Authors:** Faisal S AlSuliman, Mohamed S Zaazouee

**Affiliations:** 1 Dentistry, King Fahad Medical City, Riyadh, SAU; 2 Faculty of Medicine, Al-Azhar University, Assuit, EGY

**Keywords:** dental care, cross-sectional, anxiety, depression, mental health, oral health

## Abstract

Background

Mental disorders cause psychological stress and lead to poor lifestyle behaviors and an increased risk of poor oral health. This study aims to explore the potential association between mental illnesses with oral health and personal oral care in the Saudi population.

Methodology

Saudi Arabians aged ≥18 years were eligible to participate in this cross-sectional study. The study questionnaire had the following five sections: demographics, a brief depression severity measure (Patient Health Questionnaire-9), a brief generalized anxiety disorder measurement tool (Generalized Anxiety Disorder-7), an oral health measurement tool, and personal oral health care. The data were analyzed using SPSS software version 26 (IBM Corp., Armonk, NY, USA). The results were presented as numbers and (percentages) or mean and standard deviations (SD).

Results

This study included a total of 522 participants. The mean score for dental health and care was 4 (SD = 1.9) and 13.6 (SD = 1.9), respectively, reflecting a moderate level of dental health and positive dental care. Males had better oral health, whereas females had better dental care. A college degree or higher was linked to better dental care, and chronic diseases were linked to lower dental health scores. Minimal depression had a significantly higher dental care score than mild-to-severe depression. Depression and anxiety did not affect dental health.

Conclusions

This study showed that minimal depression was associated with a higher dental care score than mild-to-severe depression. However, the degree of depression was not associated with dental health. Furthermore, anxiety had no association with dental health or care.

## Introduction

According to the World Health Organization (WHO), mental disorders are characterized by a clinically significant disturbance in an individual’s cognition, emotional regulation, or behavior [1]. Mental disorders are common; as of 2019, one in every eight people in the world, or about 970 million people, were living with a mental disorder. The most common disorders are anxiety and depressive disorders [2]. In 2020, mostly due to the coronavirus disease 2019 pandemic, there was a significant increase in this number, with initial estimates of 26% and 28% annual increase for anxiety and major depressive disorders, respectively [3].

Mental disorders negatively impact physical health [4-7]. Previous studies have discussed the association between mental disorders and chronic physical health conditions such as cancers, heart diseases, and obesity [8-11]. However, minimal concern was targeted toward the association between mental health and oral health [12]. Worldwide, oral health issues, including untreated dental caries, periodontal diseases, and orodental trauma, are prevalent, affecting about 3.5 billion people [12,13]. A previous systematic review estimated that, among Saudi children, the prevalence of dental caries was 80% for primary dentition and 70% for permanent dentition [14].

People with mental disorders suffer more from psychological stress and poor lifestyle behaviors [15-17]. Consequently, they have an increased risk of poor oral health [18-23]. One of the basic human rights is access to healthcare. This is specifically important when referring to a vulnerable population as stigma and discrimination can come in the way of easy access to healthcare [24]. There is limited data regarding the association between dental and mental health in the Saudi population. Therefore, this study aims to test the presence of a potential association between oral health and personal oral health care as outcomes and mental illnesses (depression and anxiety) as exposure in the Saudi population.

## Materials and methods

Study design and setting

This was an online survey-based cross-sectional study conducted through online and social media platforms of the Saudi population from May 01, 2021, to August 15, 2021. The study was conducted and reported in accordance with the Strengthening the Reporting of Observational Studies in Epidemiology (STROBE) guidelines. Institutional Review Board approval was obtained from King Abdulaziz City for Science and Technology, Kingdom of Saudi Arabia (H-01-R-012).

Study participants

The eligibility criteria required that participants be Saudi Arabians exceeding the age of consent (18 years). There were no further restrictions in terms of age, gender, or demographics. On the other hand, people living in Saudi Arabia but of different nationalities were excluded because of potentially different demographic, social, and financial situations.

Sample size and allocation strategy

We used the convenience sampling method to collect data from the population via online survey distribution. The sample size was calculated for each age group in the country separately using the equation n = z2P(1-P)/d2 [25]. Under a 95% confidence interval (CI), 50% response, and a 0.05 margin of error, a sample of 384 participants was considered a minimal sample representing big populations.

Study questionnaire

The study used a five-section questionnaire to measure the variables of interest. The five sections included a demographics section, a validated Arabic version of the brief depression severity measure (Patient Health Questionnaire-9, PHQ-9), a validated Arabic version of the brief generalized anxiety disorder measurement tool (Generalized Anxiety Disorder-7, GAD-7), an oral health measurement tool, and personal oral health care. The questionnaire is provided in the Appendices.

Scoring of the Dental Health and Dental Care Questionnaires

The seven items of dental health parameters were given a score of 1 if they were not present or 0 if they were present for each participant, yielding a score ranging from 0 to 7, with higher scores indicating better dental health.

The six items of the dental care questions were given a score of 1 to 3 according to the following: the most optimum dental care practices were given a score of 3, less effective practices were given a score of 2, and non-effective or harmful practices were given a score of 1, yielding a score ranging from 6 to 18, with higher scores indicating better dental care practices.

Statistical analysis

All data were analyzed using SPSS software version 26 (IBM Corp., Armonk, NY, USA). Means and standard deviations (SDs) were used to describe the scores of both dental health and dental care. Frequency and percentage were used to describe the demographics as well as the individual items of the dental health and dental care questionnaires.

To test the association between dental health, dental care, and demographics, the t-test was used to compare the scores among different demographic groups. To test the association between dental health, dental care scores, and mental health measures (depression and anxiety), the one-way analysis of variance test was used. A p-value <0.05 was considered statistically significant.

## Results

Demographic characteristics of the study sample

This study included 522 participants, of which 41.6% were male, 69.3% had a college degree or above, and 62.5% were single. Complete demographic characteristics are presented in Table 1.

**Table 1:** Demographic characteristics of the study participants. Data are presented as numbers and percentages (%) except for age which is presented as mean and standard deviation (SD).

Basic characteristics		Total
Age		29.2 (10.3)
Gender	Male	217 (41.6%)
	Female	305 (58.4%)
Education	College or above	362 (69.3%)
	Below college	160 (30.7%)
Marital status	Single	326 (62.5%)
	Married	196 (37.5%)
Having a chronic disease	Yes	190 (36.4%)
	No	332 (63.6%)
Smoking	Yes	41 (7.9%)
	No	481 (92.1%)
Alcohol intake	Yes	6 (1.1%)
	No	516 (98.9%)
Body mass index	Underweight	44 (8.4%)
	Normal	243 (46.6%)
	Overweight	127 (24.4%)
	Obese	107 (20.5%)

Level of dental health and dental care practices among the study participants

For dental health, with a score ranging from 0 to 7, the mean score of the study sample was 4 (SD = 1.9), reflecting a moderate level of dental health. Major gaps in the reported dental health included the level of dental caries (53.3%), dental filling (65.7%), and loss of any permanent teeth (47.4%) (Table 2).

**Table 2:** Frequency and percentage of different dental health items. Data are presented in numbers and percentages (%).

Item	Yes	No
Dental caries	262 (53.3%)	230 (46.7%)
Loss of any permanent teeth	233 (47.4%)	259 (52.6%)
Dental filling	323 (65.7%)	169 (34.3%)
Gum or teeth bleeding	220 (44.7%)	272 (55.3%)
Loose permanent teeth	83 (16.9%)	409 (83.1%)
Dental prosthesis	188 (38.2%)	304 (61.8%)
Non-replaced lost permanent teeth	188 (38.2%)	304 (61.8%)

For dental care practices, with a score ranging from 6 to 18, the mean score of the study sample was 13.6 (SD = 1.9), reflecting a moderate level of positive dental care practices. The main gaps in dental care practices included a lack of prophylactic/preventive dentist visits (10.8%), with most of the study sample only visiting a dentist when having a dental problem that affected their life (76.8%). Only 30.7% of the sample used highly effective methods of tooth brushing such as vertical brushing and Bass modified technique, with most of the participants using less effective or non-effective methods (Table 3).

**Table 3:** Frequency and percentage of different dental care practices. Data are presented in numbers and percentages (%).

Item	Best practice	Less effective practice	Non-effective or harmful practice
Number of times of tooth brushing per day	200 (40.7%)	273 (55.5%)	19 (3.9%)
Duration of teeth brushing per day	199 (40.4%)	279 (56.7%)	14 (2.8%)
Frequency of toothbrush replacement	210 (42.7%)	262 (53.3%)	20 (4.1%)
Frequency of dentist visits	53 (10.8%)	378 (76.8%)	61 (12.4%)
Method of tooth brushing	151 (30.7%)	325 (66.1%)	16 (3.3%)
Extra methods for mouth and teeth cleaning	212 (43.1%)	186 (37.8%)	94 (19.1%)

Association between levels of dental health and dental care practices with the demographic characteristics

The male gender was associated with better dental health, while the female gender was associated with higher dental care practice scores. Having a college degree or above was associated with better dental care practices, and having a chronic disease was associated with lower dental health scores. Participants in the obesity range of the body mass index (BMI) had significantly lower dental health and dental care scores than those with normal BMI (Table 4).

**Table 4:** Association between dental health/dental care and demographics. SD: standard deviation

Demographic		Dental health score, mean (SD)	P-value	Dental care practices score, mean (SD)	P-value
Gender	Male	4.3 (1.9)	0.003	13.1 (2.2)	<0.001
	Female	3.8 (1.8)		13.9 (1.7)	
Marital status	Single	4.4 (1.8)	<0.001	13.6 (2)	0.6
	Married	3.4 (1.8)		13.7 (1.9)	
Education	Below college	4.1 (2)	0.6	13.3 (2)	0.04
	College or above	4 (1.8)		13.7 (1.8)	
Smoking	Yes	3.9 (1.9)	0.8	13.1 (2.2)	0.1
	No	4 (1.9)		13.7 (1.9)	
Body mass index	Underweight	4.3 (1.9)	0.03	13.9 (1.9)	0.02
	Normal	4.2 (1.7)		13.8 (1.7)	
	Overweight	3.9 (2.1)		13.6 (1.8)	
	Obese	2.5 (1.9)		13 (2.3)	
Chronic disease	Yes	3.7 (1.9)	0.003	13.7 (1.8)	0.3
	No	4.2 (1.8)		13.5 (2)	

Association between dental health/dental care and mental health (depression and anxiety)

Participants with minimal state of depression had significantly higher dental care practice scores than those who had mild-to-severe depression. The degree of depression was not significantly associated with dental health. The degree of anxiety among the participants was not significantly associated with their dental health or dental care practices in the study sample (Table 5).

**Table 5:** Association between dental health/dental care and both depression and anxiety (analysis of variance test). SD = standard deviation

Demographic		Dental health score, mean (SD)	P-value	Dental care practices score, mean (SD)	P-value
Depression	Minimal	4.1 (1.9)	0.8	14.2 (1.7)	0.002
	Mild	4 (1.9)		13.5 (1.9)	
	Moderate	3.9 (1.9)		13.4 (1.9)	
	Moderately severe	4 (1.8)		13.5 (2.1)	
	Severe	3.8 (1.9)		13.1 (2.4)	
Anxiety	Minimal	4 (1.9)	0.48	13.8 (1.9)	0.18
	Mild	4.2 (1.9)		13.6 (1.8)	
	Moderate	4 (1.9)		13.6 (1.8)	
	Severe	3.6 (1.8)		13.1 (2.4)	

## Discussion

Although the study found a moderate level of dental health among participants, the major gaps included dental filling, dental caries level, and loss of any permanent teeth. Further, the participants had a moderate level of positive dental care practices, while the main gaps included a lack of prophylactic/preventive dentist visits. Moreover, males were associated with better dental health, but females were associated with higher dental care practices. Having a college degree or above was associated with better dental care practices, while having a chronic disease was associated with lower dental health scores. Participants suffering from obesity had significantly lower dental health and dental care scores than those with normal BMI.

Regarding the association between dental health/dental care and mental health, the study found that participants with minimal state of depression had significantly higher dental care practice scores than those who had mild-to-severe depression. On the other hand, the severity of the depression was not significantly associated with dental health. The degree of anxiety was not significantly associated with dental health or dental care practices.

Delgado-Angulo et al. previously found that, among Finnish people, depression was significantly associated with decayed teeth among participants aged 35-54 years, unlike other age groups [21]. However, both depression and anxiety were not significantly related to periodontal disease. A cross-sectional study of 5,900 participants in Iran showed a significant association between depression and oral health indices but not with anxiety, which highlighted the need for more attention on oral health among those with a history of depression [26].

On the other hand, a cross-sectional study conducted in Spain among 23,089 participants showed a positive association between any psychiatric condition and poor oral health outcomes, as well as a significant association between any psychiatric condition and marital status, with marriage showing protective benefits [12]. A previous meta-analysis of 26 studies found that all psychiatric diagnoses were associated with increased dental decay on both Decayed, Missing, and Filled Teeth (DMFT) and Decayed, Missing, and Filled Surfaces (DMFS) scores, and greater tooth loss. On the other hand, no association was found with periodontal disease apart from panic disorder [27].

Torales et al. concluded that it is crucial to be aware of the common issues in the population among those suffering from mental illnesses. This is because they are vulnerable groups for various reasons, including lack of motivation and oral hygiene, fear of visiting the dentist, difficulty in accessing health services, and side effects of medications, particularly xerostomia [28].

This study highlights the need for offering more attention and assistance to people suffering from mental illnesses in Saudi Arabia, specifically depression, concerning dental care practice. Examples of these interventions include launching campaigns to support the elimination of any form of stigma mental disorder patients face as well as support easier dental care access in the form of dentists spreading more awareness and educating the public regarding this matter. Even though this study showed no significant association between depression and anxiety with dental health, conclusive findings could not be made as more data with larger sample sizes are needed. Moreover, the association between dental health and mental health requires further investigation.

The limitations of this study include collecting data through an online questionnaire which increases the possibility of participation bias. Because our study was cross-sectional, the temporal relevance of our findings may vary over time or with the use of large-scale preventative interventions.

## Conclusions

The study showed that minimal depression was associated with significantly higher dental care practice scores than mild-to-severe depression. However, the degree of depression was not significantly associated with dental health. Further, the degree of anxiety was not significantly associated with dental health or dental care practices.

## Appendices

Associations between mental health and oral health in Saudi Arabia: an online Survey

The main objective of the study is to test the presence of a potential association between mental illnesses (depression and anxiety) with oral health and personal oral health care in the Saudi population. The questionnaire consists of five sections aimed at measuring demographic information, depression, anxiety, oral health, and the extent of interest in oral and dental health, in order.

Participation in this study is voluntary. The information will be used solely for research purposes. It will only be viewed by the research team. There will be no collected information that may disclose the participant’s identity.

1. This survey is voluntary. You may decline to answer any of the questions. By checking the box below, you are acknowledging that you are voluntarily participating in this survey, have read, and understood the Information Sheet, and consent.

I consent

I do not consent

2. Are you a Saudi Arabia citizen?

Yes

No

Section 1: Demographic characteristics

- Age

The value must be a number

- Gender

Female

Male

- Marital status

Single

Married

Widowed

Divorced

-  Are you a smoker?

Yes

No

Ex-smoker

- Have you drunk alcohol in the last year?

Yes

No

- What is your educational level?

High school or less

Undergraduate or more

- Weight in kilogram (approximate)

The value must be a number.

- Height in centimeters (approximate)

The value must be a number.

- Did you have any of these pre-existing conditions (you can choose more than one)?

Hypertension

Cardiomyopathy

Angina pectoris

Varicose veins

Chronic back pain

Chronic neck pain

Chronic allergy

Asthma

Chronic obstructive pulmonary disease (COPD)

Diabetes

Gastric ulcer

Urine incontinence

Hyperlipidemia

Chronic skin disease

Chronic constipation

Liver cirrhosis

Stroke

Migraine

Piles

Osteoporosis

Cancer

Thyroid diseases

Kidney diseases

Others (mention them)

Section 2: A brief depression severity measure (PHQ-9)

Over the last two weeks, how often have you been bothered by any of the following problems?

**Table 6:** A brief depression severity measure (PHQ-9). PHQ-9: Patient Health Questionnaire-9

Question	Not at all	Several days	More than half the days	Nearly every day
Little interest or pleasure in doing things				
Feeling down, depressed, or hopeless				
Trouble falling or staying asleep or sleeping too much				
Feeling tired or having little energy				
Poor appetite or overeating				
Feeling bad about yourself—or that you are a failure or have let yourself or your family down				
Trouble concentrating on things, such as reading the newspaper or watching television				
Moving or speaking so slowly that other people could have noticed? Or the opposite—being so fidgety or restless that you have been moving around a lot more than usual				
Thoughts that you would be better off dead or hurting yourself in some way				

Section 3: A brief generalized anxiety disorder measurement tool (GAD-7).

Over the last two weeks, how often have you been bothered by the following problems?

**Table 7:** A brief generalized anxiety disorder measurement tool (GAD-7). GAD-7: Generalized Anxiety Disorder-7

Question	Not at all	Several days	More than half the days	Nearly every day
Feeling nervous, anxious, or on edge				
Not being able to stop or control worrying				
Worrying too much about different things				
Trouble relaxing				
Being so restless that it is hard to sit still				
Becoming easily annoyed or irritable				
Feeling afraid as if something awful might happen				

Section 4: Dental health items

**Table 8:** Dental health items.

Item	Yes	No
Do you have dental caries?		
Have you extracted any of your permanent teeth?		
Do you have a dental filling?		
Do you suffer from bleeding gums with/without brushing your teeth?		
Do you suffer from loosening or movement of any of your permanent teeth?		
Do you have any prostheses in your teeth? (crowns, bridges, or other)		
Do you have any extracted/missing teeth that have not been replaced by dentures?		

Section 5: Dental care practices

How many times do you brush your teeth daily?

Once or less

Twice

Three times

More than three times

I don’t brush my teeth

2- How long do you brush your teeth at once?

A minute or less

Two minutes

Three minutes or more

I don’t brush my teeth

When do you change your toothbrush?

Every three months or less

Every half year

Change it only when it is completely unusable

I don’t brush my teeth

When do you visit the dentist?

Periodically

When I suffer from a disease in my teeth

When I suffer from a dental disease for a long time

When my quality of life is disrupted by illness

Never go to a dentist

How do you brush your teeth?

Vertical brushing

Horizontal brushing

Modified “bass” method

Fones method

Irregularly in all directions

I don’t brush my teeth

What other oral and dental cleaning methods do you use besides brushing?

Dental floss

Mouthwash

Sugar-free chewing gum

Toothpicks (Miswaak)

Teeth cleaning pick

Nothing
